# Relevance of Immunohistochemistry for Tumorigenic Tumor-Infiltrating Neutrophils and Reverse Polarity in Colonic Micropapillary Adenocarcinoma: A Case Report

**DOI:** 10.1155/crip/9365437

**Published:** 2025-08-04

**Authors:** Kazumori Arai, Kensuke Shimazaki, Koji Takahashi, Hiroyuki Hazama, Ko Ohata, Akihiro Sonoda, Tomohiro Iwasaki, Junichi Sakane

**Affiliations:** ^1^Department of Pathology, Shizuoka General Hospital, Shizuoka, Japan; ^2^Department of Colorectal Surgery, Shizuoka General Hospital, Shizuoka, Japan; ^3^Department of Clinical Research, Shizuoka General Hospital, Shizuoka, Japan

## Abstract

Similar to that in other organs, colorectal micropapillary adenocarcinoma (MPA) shows aggressive biological characteristics and reverse polarity (RP). Inhibiting the RP may reduce cancer aggressiveness; however, the pathogenesis of RP remains unclear. We encountered a case of colorectal MPA with tumor-infiltrating neutrophils (TINs), which were suspected to be involved in micropapillary morphogenesis. We examined the case using immunohistochemistry, including luminal differentiation (LD) markers. Numerous TINs were found within the background MPA tumor components, and there were scattered tumor cell detachments from the stroma and disruption of glandular structures. Furthermore, the ruptured lumens were connected to the lacunar stromal spaces created by the tumor cell detachment, and floating isolated tumor cell clusters were observed. Immunohistochemistry suggested that most of the TINs had immunosuppressive and tumor-promoting properties and that the tumor cells that have lost adhesion to the stroma and/or intercellular contacts acquired new LD. Such tumor cell changes have been observed in our previous report on tumors with frequent apoptosis. Based on this case, we suggested that (1) the essence of RP in MPA comprises new LD/apical polarity in tumor cells, which have lost glandular polarity secondary to exfoliative and destructive changes, and (2) the cause of RP might be multifactorial.

## 1. Introduction

Micropapillary adenocarcinoma (MPA) of the colon is characterized by tumor cell clusters that are devoid of fibrovascular cores and float in lacunar stromal spaces resembling dilated lymphatic vessels [1–3]. The incidence of this unique tumor in the colorectum ranges from 9% to 19% [2]. However, pure MPA is rare and usually coexists with the components of conventional adenocarcinoma [2, 3]. Colorectal MPA, like that of other organs, is known to show aggressive biological characteristics, such as lymphovascular invasion and nodal metastasis, and generally has a poor prognosis [1–3]. Therefore, a micropapillary (MP) component that is > 5% of the entire tumor, or even < 5%, according to some researchers, is considered pathologically significant [1, 3]. Furthermore, regardless of the primary organ, a major characteristic of MPA is reverse polarity (RP), which means that the stroma-facing surfaces of the tumor cells show luminal differentiation (LD). Inhibiting the RP in adenocarcinoma may reduce cancer aggressiveness; however, the pathogenesis of RP remains unclear [3, 4].

MP structures are considered one of the putative apoptosis-resistant cell subpopulations in colorectal adenocarcinoma [5]. We have previously reported an unusual case of colonic MPA with frequent apoptosis [6]. In that case, we suggested the relationship between the impairment of tumor cell–stroma adhesion and apical–basal polarity in tumor cells due to apoptosis and RP [6]. Considerably, MP morphogenesis is caused by various pathological changes that disrupt glandular polarity [6, 7]. Herein, the relationship between tumor-infiltrating neutrophils (TINs) and MP morphogenesis in a colorectal MPA with abundant TINs was investigated immunohistochemically.

## 2. Case Presentation

A 53-year-old woman was referred to our hospital for further evaluation of occult fecal blood in a routine physical. Past medical history was unremarkable and excluded cancer; however, she had a family history of renal cancer, which was experienced by her mother. Neither fever nor weight loss was present, and defecation frequency was normal. Before visiting our hospital, the patient used to smoke five cigarettes and drink two small bottles of beer every day for 33 years.

Various imaging procedures, including computed tomography, revealed no metastases to the lymph nodes or other organs. Tumor markers were within normal ranges: carcinoembryonic antigen was at 2.2 ng/mL (normal, 0–5 ng/mL) and cancer antigen 19-9 was at 6.0 U/mL (normal, 0–37 U/mL). Colonoscopy revealed a protruding tumor with central depression, occupying almost half of the circumference of the transverse colon. Biopsy of this tumor revealed adenocarcinoma. Subsequently, robot-assisted laparoscopic partial colectomy and lymph node dissection were performed. There was no mismatch repair deficiency on immunohistochemical screening. No analysis for Ki-ras2 Kirsten rat sarcoma viral oncogene homolog (*KRAS*) mutation status was performed. After surgery, the patient received eight cycles of oxaliplatin combined with capecitabine for 6 months and did not have recurrence during the follow-up period of 17 months after surgery.

### 2.1. Pathological Examinations

The tumor sample was fixed in 10% neutral buffered formalin, and paraffin-embedded five tissue sections were routinely stained with hematoxylin and eosin. We defined the invasive tumor components associated with MPA as background components (BGCs) because they directly underlie MPA development. Accordingly, we focused on BGCs, as they were considered more appropriate than completed MPA components for examining the relationship between TINs and MP morphogenesis [6, 7].

Naphthol AS-D chloroacetate esterase plus Giemsa double staining [8] was performed to identify neutrophils. The number of TINs was measured only for neutrophils within and in close proximity to the tumor cells semiquantitatively [9]. In the BGCs, the numbers of TINs per 100 tumor cells were measured at 20 high-power fields (×40 objective lens with a field area of 0.307 mm^2^) in the area with the most prominent TIN infiltration, and the median value was calculated [9]. The median was then compared with that of the invasive tumor components not accompanied by any MPA components, that is, the non-BGCs, which were measured using the same method.

Serial tissue sections from three representative tissue blocks, including the tumor center and margin, were examined by immunohistochemistry using the Leica Bond-Max (Leica Biosystems, Melbourne, Australia). All the antibodies used are listed in Table 1. Podoplanin (D2-40) is a frequently used marker to distinguish between lymphatic vessels and lacunar stromal spaces [6]. Cluster of differentiation (CD) 10 and lectin-type oxidized low-density lipoprotein receptor 1 (LOX1) are neutrophil-related markers [10, 11]. Phosphorylated histone variant H2AX (Ser139) (*γ*H2AX) is an apoptosis-related marker [12]. Epithelial membrane antigen (EMA), mucin 1 glycoprotein (MUC1), and Annexin A2 (ANX A2) are LD markers [2–4, 6, 7, 13–15]. CD10-positive TIN and LOX1-positive TIN counts were also evaluated using the same method.

### 2.2. Pathological Findings

**Figure 1:**
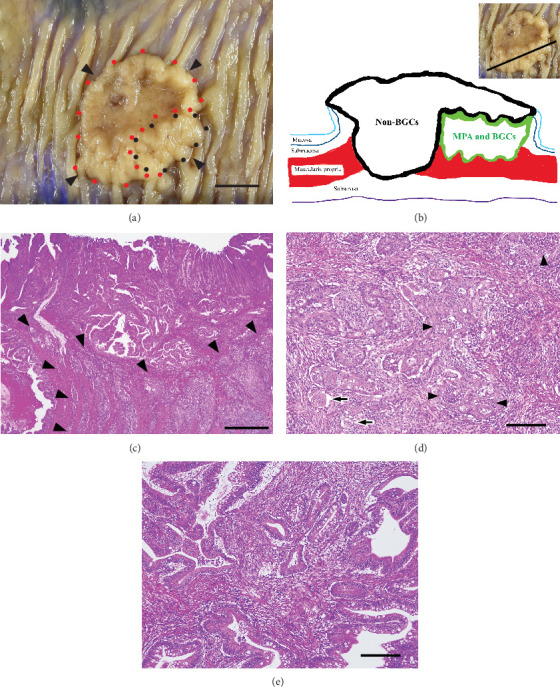
Pathological findings. (a) On gross view, the tumor was a protruding tumor with mild central depression (surrounded by arrowheads). Areas containing the background components (BGCs) of micropapillary adenocarcinoma (MPA) components on histology are outlined in black, whereas non-BGC areas are outlined in red (scale bar, 1 cm). (b) Simplified schematic of the cross-section of an MPA, corresponding to the cutting line in the inset, indicating the positional relationship of the tumor with the BGC and non-BGC areas. MPA and the BGCs, which are localized in the protruding section of the tumor, are present in the submucosa to muscularis propria and covered by the non-BGC area. MPA and the BGC area are surrounded by a thick green line, whereas the non-BGC area is surrounded by a thick black line. (c) Histological examination demonstrates adenocarcinoma, comprising mainly components with a distinct papillary or tubular growth pattern but with some invasive tumor components with indistinct papillary and tubular structures (corresponding to the BGCs) in the submucosa and deeper layers (surrounded by arrowheads) (H&E stain; scale bar, 1 mm). (d) A fivefold magnified image of the BGCs surrounded by the arrowheads in (b). Irregular cribriform pattern with severe inflammatory cell infiltrates, lumens filled with neutrophils (arrowheads), and MPA components (arrows) are seen (H&E stain; scale bar, 200 *μ*m). (e) Compared with the BGCs, the non-BGCs have less inflammatory cell infiltration (H&E stain; scale bar, 200 *μ*m). GCs, background components; H&E, hematoxylin and eosin; MPA, micropapillary adenocarcinoma.

Figure 1 shows the pathological findings. Macroscopic examination revealed a protruding tumor with mild central depression, measuring 3 × 2.8 cm. Microscopically, the tumor was adenocarcinoma and had invaded up to the subserosa. The tumor metastasized to three regional lymph nodes, corresponding to pathological stage IIIB (pT3N1bM0) [16]. Main tumor components showed only distinct papillary and tubular structures. However, the tumor components with a primarily irregular cribriform pattern were locally seen in the submucosa and deeper layers, corresponding to the proximal side of the protrusion. These components represented approximately 30% of the total tumor volume, accompanied by MPA components, and were considered the BGCs. The MPA components represented only > 5% of the total tumor volume and were located outside the main tumor components.

The BGCs were further accompanied by severe inflammatory cell infiltrates compared with main tumor components, that is, the non-BGCs. The inflammatory cells included abundant neutrophils within and/or in close proximity to the tumor cells. Semiquantitative measurements [9] and Wilcoxon's signed-rank test using Jamovi (Version 2.5.3) (https://www.jamovi.org/) showed that the number of TINs per 100 tumor cells was significantly higher in the BGCs than in the non-BGCs (61.6 [IQR, 59.1–71.9)] vs. 1.5 [IQR, 0.3–2.2], *p* < 0.001). No significant inflammation was seen in the nontumor colon wall, and little neutrophilic infiltration was observed.

**Figure 2:**
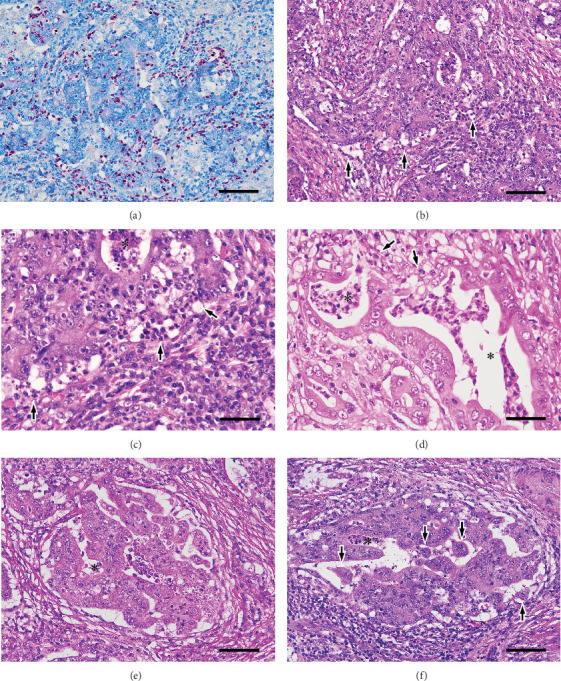
Pathological findings of the background components. (a) Several neutrophils with reddish cytoplasm are concentrated within and/or in close proximity to the tumor cells (naphthol AS-D chloroacetate esterase plus Giemsa double stain; scale bar, 100 *μ*m). (b) Along with severe neutrophil infiltration, there is scattered tumor cell detachment from the stroma, causing the formation of cleft-like spaces on the periphery of the tumor cell nests (H&E stain; scale bar, 100 *μ*m). (c) An image of cleft-like spaces in (b) magnified two times. Numerous neutrophils infiltrate and extend into the tumor cells and lumen (asterisk) of the tumor gland (H&E staining; scale bar, 50 *μ*m). (d) In some tumor glands, the lumens (asterisks) are directly connected to the stroma (arrow), indicating the ruptured lumens, that is, tumor gland destruction (H&E staining; scale bar, 50 *μ*m). (e) Irregularly shaped spaces are seen inside and outside the tumor cell nest because of tumor cell detachment and disruption of glandular structures (H&E staining; scale bar, 100 *μ*m). An asterisk indicates the lumen of the tumor gland. (f) Small tumor cell clusters (arrows) from the destructed tumor glands are floating in the dilated space, which is connected to the ruptured lumen (asterisk), or in the stromal space, and form a microcapillary pattern (H&E staining; scale bar, 100 *μ*m). H&E, hematoxylin and eosin.

Details of the pathologic examination of the BGCs and numerous TINs are shown in Figure 2. Tumor cell detachment from the stroma was scattered, and lumens of the tumor glands directly connected to the stroma; the latter was interpreted as ruptured lumens, that is, tumor gland destruction. Furthermore, MP patterns were seen in the tumor cell nests that exhibited such changes. Apoptotic tumor cells were hardly identified in the BGCs.

### 2.3. Immunohistochemical Findings

#### 2.3.1. Assessment of Lymphatic Vessels and Neutrophil-Related Markers

**Figure 3:**
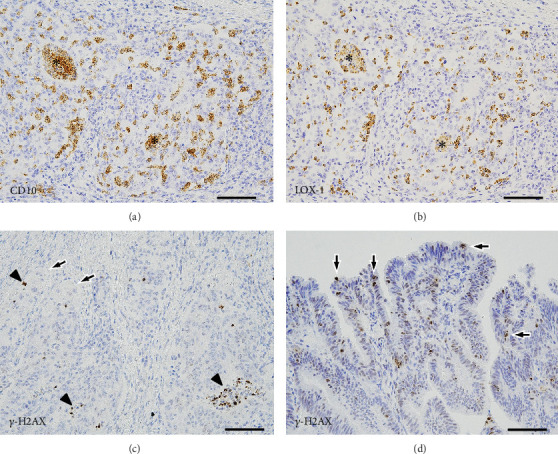
Immunohistochemical findings. Photomicrographs of the background components (BGCs) show several (a) CD10-positive and (b) LOX1-positive cells, which are concentrated within and/or in close proximity to the tumor cells (asterisks indicate the lumens of the tumor glands). (c) On *γ*H2AX immunostaining, most of the nuclei with strong positivity are present within the lumens (arrowheads) and lacunar stromal space, which represent apoptotic neutrophils, and there are no distinct positive tumor cells, including micropapillary adenocarcinoma components (arrows). (d) Photomicrograph of the non-BGCs shows scattered *γ*H2AX-positive tumor cells (arrows) with varying staining intensities on the tumor surface (scale bars, 100 *μ*m). BGCs, background components; LOX1, lectin-type oxidized low-density lipoprotein receptor 1; *γ*H2AX, phosphorylated histone variant H2AX (Ser139).

Many of the lacunar stromal spaces were negative for D2-40, indicating the absence of lymphatic vessels. For the neutrophil-related markers (Figure 3a,b), most of the TINs were CD10-positive. Overwhelmingly, the number of tumor-infiltrating CD10-positive cells per 100 tumor cells was significantly higher in the BGCs than in the non-BGCs (59.5 [IQR, 55.6–69.4] vs. 0.4 [IQR, 0.3–2.0], *p* < 0.001; Wilcoxon's signed-rank test). Moreover, many of the TINs were LOX1-positive, with a similar and overlapping distribution with the CD10-positive cells. Likewise, the number of tumor-infiltrating LOX1-positive cells per 100 tumor cells was significantly higher in the BGCs than in the non-BGCs (55.9 [IQR, 44.9–65.8] vs. 0.3 [IQR, 0.1–3.5], *p* < 0.001; Wilcoxon's signed-rank test). Neither CD10-positive nor LOX1-positive neutrophils were found in the submucosa or deeper layers of the nontumor colon wall.

#### 2.3.2. Analysis of the Apoptosis-Related Marker

As shown in Figure 3c,d, there were few or no distinct *γ*H2AX-positive tumor cells in the BGCs. Most of the strongly *γ*H2AX-positive nuclei were present within the lumens or lacunar stromal spaces, representing apoptotic TINs. However, in the non-BGCs, cells with varying *γ*H2AX staining intensities were scattered primarily on the tumor surface.

#### 2.3.3. Analysis of the LD Markers in BGCs

**Figure 4:**
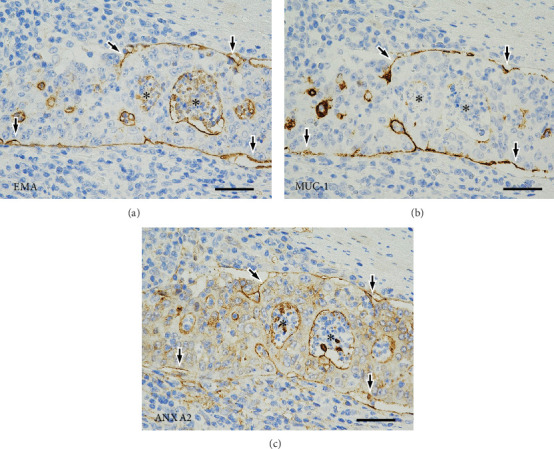
Immunohistochemical findings in the Background Components I. On immunostaining with the three luminal differentiation markers (a) EMA, (b) MUC1, and (c) ANX A2, serial tissue sections of tumor cell nests with detachment from the stroma show linear positivity on the stroma-facing tumor cell surfaces (arrows). Asterisks indicate the lumens of the tumor glands (scale bars, 50 *μ*m). EMA, epithelial membrane antigen; MUC1, mucin 1 glycoprotein; ANX A2, Annexin A2.

**Figure 5:**
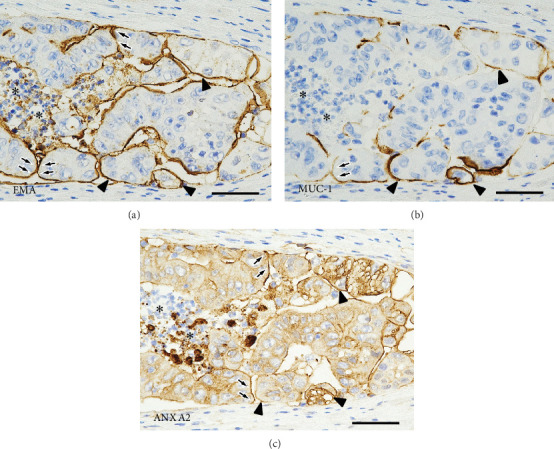
Immunohistochemical findings in the Background Components II. On immunostaining with the three luminal differentiation markers (a) EMA, (b) MUC1, and (c) ANX A2, serial tissue sections of disrupted tumor glands that are detached from the stroma show that the positive reactions on the lumen and the side facing the stroma are connected (arrows). In the small clusters of tumor cells, the positive reactions are seen on the surface cell membranes, partially or circumferentially (arrowheads). Irregularly shaped spaces connected to the lumen (asterisks) of the tumor gland and cleft-like stromal spaces indicate tumor gland destruction (scale bars, 50 *μ*m). EMA, epithelial membrane antigen; MUC1, mucin 1 glycoprotein; ANX A2, Annexin A2.

**Figure 6:**
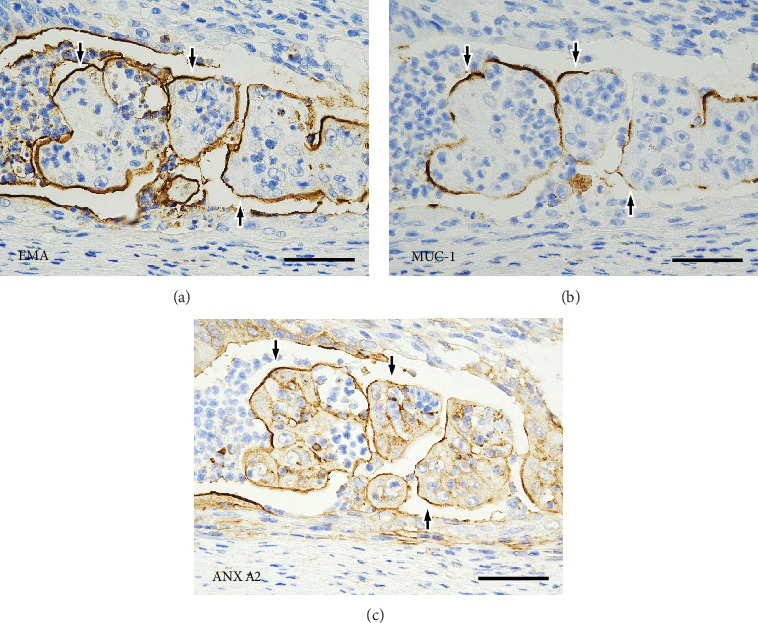
Immunohistochemical findings in micropapillary carcinoma components. Serial tissue sections on immunostaining with the three luminal differentiation markers (a) EMA, (b) MUC1, and (c) ANX A2 show linear positivity on the surfaces of the tumor cell clusters, partially or circumferentially (arrows) (scale bars, 50 *μ*m). EMA, epithelial membrane antigen; MUC1, mucin 1 glycoprotein; ANX A2, Annexin A2.

Figure 4 describes the LD in the tumor cell nests with detachment from the stroma. Staining for EMA and MUC1 showed a linear positivity along the stroma-facing surfaces of the tumor cells. Positivity for ANX A2 showed a similar and overlapping pattern. Further details of the staining patterns of the LD markers in the disrupted tumor glands and in the MPA components are shown on Figures 5 and 6, respectively.

## 3. Discussion

In solid cancers, there is often an abundance of infiltrating neutrophils [17]. TINs have a dual function of being antitumorigenic and tumorigenic [11, 17–21]. Furthermore, they have the plasticity to change phenotypes and functions, depending on various factors, such as cytokines and chemokines, which are derived from tumor cells and constitutive cells of the tumor environment [11, 17–21]. In the present case, most of the TINs were positive for CD10 and LOX1. CD10 is specifically expressed by mature neutrophils in the latest stages of differentiation [10]. LOX1 is overexpressed in polymorphonuclear myeloid–derived suppressor cells (PMN-MDSC) [11, 22] and enhances reactive oxygen species (ROS) production [22]. Recently, LOX1-positive TINs were described to possess MDSC activity and suppress antitumor immune response [21, 22]. Most of the TINs in the present case are considered to be mature neutrophils with tumorigenic PMN-MDSC activity [11, 21, 22]. Mature neutrophils with MDSC activity are well known for their properties of ROS production and degranulation [20, 21, 23, 24]. ROS and proteinases released by neutrophils damage tumor cell membranes and the stroma, including the extracellular matrix [11, 20, 24], potentially disrupting tumor cell–stroma adhesion [11, 20, 24, 25]. This disruption may lead to tumor cell detachment from the stroma and tumor cell-to-cell separation, thereby triggering the loss of apical–basal cell polarity [13, 14, 26, 27]. If this adhesion loss progresses and detached tumor cells acquire new LD, RP may occur and eventually form MPA components [13, 14, 26, 27].

EMA and MUC1 were the gold standard markers for LD [2–4, 6, 7]. ANX A2 is a subtype of annexin [15, 28], which is a multigene family of calcium-signaling and membrane-bound proteins. ANX A2 is deposited in cell membranes, which eventually become a lumen, and accumulates in the luminal surface to contribute to the establishment of apical polarity (AP) [13–15]. In the present case, the linear pattern of positivity for EMA, MUC1, and ANX A2 on the stroma-facing surfaces of the detached tumor cells suggested new LD/AP [6, 7, 13–15, 26, 27]. The connection of positivity between the ruptured luminal surfaces and the stroma-facing surfaces of the detached tumor cells suggested another newly acquired LD/AP on the exposed cell membranes, which resulted from loss of polarity of the intercellular contact membranes of the tumor cells [6, 7, 13–15, 26, 27].

**Figure 7:**
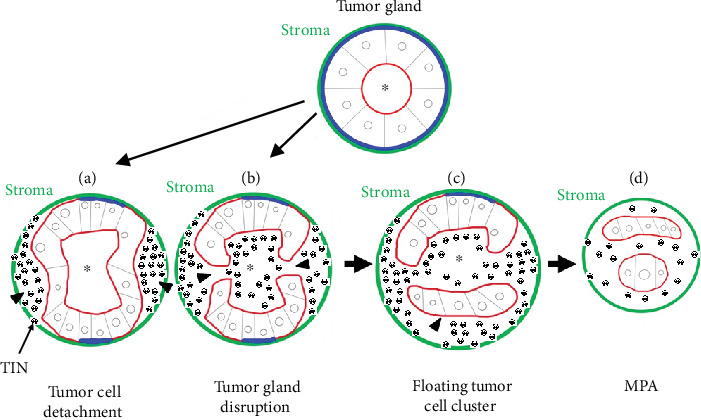
Brief schematic illustration of the reverse polarization linked with micropapillary morphogenesis by tumor-infiltrating neutrophils (TINs). (a) Tumor cell detachment from the stroma (green circle) and toward the lumen (asterisk) of the gland results in the formation of lacunar stromal spaces (arrowheads) in the periphery. The tumor cell surfaces that face the stroma (green circle) and the neighboring cells lose basal polarity (blue curves) and acquire new LD/AP (drawn in red). (b) Tumor gland disruption and tumor cell detachment from the stroma (green circle) create connections (arrowheads) between the ruptured lumen (asterisk) and the lacunar stromal spaces in the periphery. Along with the stroma-facing surfaces, the intercellular contact membrane is exposed and also acquires a new LD/AP (drawn in red). (c) Immediately thereafter, exfoliative and destructive changes in the tumor gland leads to the formation of a floating small cluster of tumor cells (arrowhead). The surfaces of the cluster that has already lost its basal polarity (blue curve) and intercellular contact acquires an LD/AP (i.e., reverse polarity; drawn in red). (d) Eventually, the typical micropapillary adenocarcinoma component comprises only small floating tumor cell clusters that show reverse polarity. LD/AP, luminal differentiation/apical polarity; TIN, tumor-infiltrating neutrophil.

In the present case, unlike our two previously reported cases, tumorigenic TINs may have played a significant role in MP morphogenesis. We considered that the initial step in MP morphogenesis involved sporadic tumor cell detachment from the stroma and destruction of tumor glands within a tumor cell nest [6, 7, 13–15, 26, 27]. Furthermore, we believe that the essence of RP in MPA is the addition of new LD/AP to tumor cells that have lost glandular polarity due to detachment and destructive changes [6, 7, 13–15, 26, 27]. Our suggestions are shown in a brief schematic illustration (Figure 7). For simplicity, RP of the tumor glands in the cribriform tumor components has been presented as a single tumor gland in Figure 7. We considered MP morphogenesis to be driven by diverse factors, that is, various pathological changes that directly and significantly affect tumor cells and the tumor microenvironment, such as numerous tumorigenic TINs and frequent apoptosis [6, 7, 11, 13–15, 17–27].

Many colonic MPAs are typically not accompanied by numerous TINs. Given this discrepancy, TINs may not drive MP morphogenesis in other MPA cases. However, the timing of pathological changes, including high TIN infiltration levels, may also be critical. Neutrophils have short half-lives and undergo rapid apoptosis [29], with many apoptotic TINs shown in Figure 3c. As neutrophilic infiltration diminishes and apoptotic cells clear, earlier inflammation or frequent apoptosis may be obscured. In contrast, cells other than neutrophils, such as macrophages and cancer-associated fibroblasts, are known to produce chemokines/cytokines and ROS as well as induce protease expression [30–32]. Furthermore, it is known that ROS induce apoptosis [30]. A recent study reported that apoptosis may mediate ROS generation [33]. In damaged tumor components, if cell polarity changes and newly acquired LD becomes irreversible and progressive even under the influence of cells other than neutrophils, then MPA components may appear even after the reduction of neutrophil infiltration. In many MPA cases, the initial cause of RP may already have been attenuated, making it difficult to identify. Therefore, pathologists may focus on cases in which MP morphogenesis has already been completed.

Herein, neutrophil apoptosis might have been involved in MP formation via ROS production [33]. However, tumor cells may acquire resistance to apoptosis early during invasion into deeper layers [6]. Thus, in contrast to our observations in two previous cases, tumor cell apoptosis might have played a negligible role in MP morphogenesis [6, 7, 34].

Suppression of MP morphogenesis has not been established. Therefore, MPA cases from which the potential causes of RP can be determined should be accumulated and analyzed. Our findings in the present case suggest that suppressing tumorigenic polarization of neutrophils via antitransforming growth factor beta blockade [17, 19, 20, 24, 29] can suppress MP morphogenesis. We propose that suppressing the influence of tumorigenic cells within the tumor environment should be considered a therapeutic strategy to prevent the development of MPA [11, 17–25, 29–33]. Inhibiting neutrophil recruitment through antichemokine/cytokine therapy [17, 19, 20, 24, 29] in combination with anti-ROS therapy [30] is a potential approach. Owing to the lack of experimental evidence, their utility should be explored in future studies.

This case report had some limitations, including its inherent limitations and the use of only CD10 and LOX1 immunohistochemistry to evaluate the nature of TINs. Furthermore, the factors that increased the number of neutrophils in the tumor cell nests were not determined. Further investigations are needed to confirm our suppositions.

In conclusion, we described a case of colorectal MPA with numerous tumorigenic TINs with MDSC activity. We suggested that the essence of RP in MPA is new LD secondary to exfoliative and destructive changes and that the cause of RP might be multifactorial. This case highlighted the fact that further research is warranted on the relationship between the tumorigenic potential of TINs and morphological and biological changes in cancer.

## Figures and Tables

**Table 1:** Antigens used for immunohistochemistry in this case.

**Antigen**	**Clone**	**Source**	**Antigen retrieval**	**Dilution rate**
Podoplanin^a^	D2-40	Nichirei Bioscience (Japan)	ER1 (20 min)	1/2
Neutrophil-related markers				
CD10	56C6	Leica Biosystems (Germany)	ER2 (20 min)	1/100
LOX1	E9C5A	Cell Signaling Technology (United States)	ER2 (10 min)	1/100
Apoptosis-related marker				
*γ*H2AX	20E3	Cell Signaling Technology (United States)	ER2 (10 min)	1/100
LD-related markers				
EMA	GP1.4	Leica Biosystems (Germany)	ER2 (30 min)	1/500
MUC1	Ma695	Leica Biosystems (Germany)	ER2 (30 min)	1/1000
ANX A2	5/Annexin II	BD Transduction Laboratories (United States)	ER1 (30 min)	1/2000

Abbreviations: *γ*H2AX, phosphor-histone H2AX (Ser139); ANX A2, Annexin A2; EMA, epithelial membrane antigen; ER1, pH 6.0 (Leica); ER2, pH 9.0 (Leica); LD, luminal differentiation; LOX1, lectin-like oxidized low-density lipoprotein receptor 1; MUC1, mucin 1 glycoprotein.

^a^Podoplanin (D2-40) is a marker commonly used to distinguish between lymphatic vessels and lacunar stromal spaces [6].

## Data Availability

The authors declare that all relevant data were included and are available in this article.
